# Extracellular vesicles related to familial hypercholesterolemia

**DOI:** 10.20945/2359-4292-2026-0022

**Published:** 2026-03-02

**Authors:** Júnea P. de P. Silvino, Cinthia E. Jannes, Rodrigo M. C. Pestana, Lucas P. de P. Silvino, Iêda de F. O. Silva, Andréa Teixeira-Carvalho, Karina B. Gomes

**Affiliations:** 1 Faculdade de Medicina, Universidade Federal de Minas Gerais, Belo Horizonte, MG, Brasil; 2 Laboratório de Genética do Instituto do Coração (InCor), Universidade de São Paulo, São Paulo, SP, Brasil; 3 Faculdade de Farmácia, Universidade Federal de Minas Gerais, Belo Horizonte, MG, Brasil; 4 Centro de Pesquisas René Rachou, Belo Horizonte, MG, Brasil

**Keywords:** Familial hypercholesterolemia, genetic variants, extracellular vesicles, cardiovascular disease

## Abstract

**Objective:**

This study aimed to evaluate extracellular vesicles (EVs) in a group of carriers of familial hypercholesterolemia (FH)-related genetic variants compared to those in family members without FH.

**Subjects and methods:**

Annexin V-positive EVs (PS^+^-EVs), cardiomyocyte-derived EVs (CardioEVs), endothelial cell-derived EVs (EEVs), platelet-derived EVs (PEVs) and tissue factor-expressing EVs (TFEVs) were evaluated to compare individuals with FH and genetic variants (n = 16) and non-FH patients without genetic variants (n = 16).

**Results:**

Increased numbers of PS^+^-EVs, CardioEVs, EEVs and TFEVs were observed in the group c arrying genetic variants. Furthermore, patients with FH who did not use statins had higher counts of these same EVs than non-FH patients who did not use statins. These EVs were significantly correlated with low-density lipoprotein cholesterol (LDL-c) levels.

**Conclusion:**

The data suggest that EVs are related to FH and that their cellular origins could be related to cardiovascular complications commonly observed in this disease.

## INTRODUCTION

Familial hypercholesterolemia (FH) is an autosomal dominant disease characterized by the presence of variants in genes related to the metabolism of low-density lipoprotein cholesterol (LDL-c). Chronic elevation of LDL-c levels predisposes patients to the risk of premature cardiovascular disease development ^(1-3)^. FH can be caused by variants located mainly in the genes encoding the low-density lipoprotein receptor (*LDLR*), apolipoprotein B (*APOB*) and proprotein convertase subtilisin/kexin type 9 (*PCSK9*), with more than 2,900 genetic alterations associated with the disease ^(4)^. FH can present multiple phenotypes because of different molecular etiologies and additional genetic factors ^(5)^, but the risk of coronary artery disease (CAD) is greater among carriers of pathogenic variants of FH ^(6)^.

The pathophysiology of atherosclerosis begins with endothelial injury, which is mediated by a cascade of intraand intercellular signaling events that shape cellular behavior within vessels and the inflammatory response ^(7)^. The main complication resulting from this process is acute coronary syndrome, which is observed more frequently in patients with FH, including young people ^(8)^. Thus, identifying plasma markers related to the atherosclerotic process, endothelial injury, and cardiac dysfunction that can identify these outcomes early in patients with FH is highly desirable.

Extracellular vesicles (EVs) 0.1-1.0 µm in diameter, also known as microparticles (MPs), originate from cell membranes in response to cell activation or apoptosis. Cells may be activated in response to diverse stress stimuli, leading to the initiation of apoptosis or alternative cellular pathways. Such activation represents a critical step for subsequent functional outcomes, including differentiation, proliferation, and programmed cell death. Unlike EVs, exosomes have endosomal origins and are vesicular bodies with membranes composed of a lipid bilayer (30-150 nm in diameter) and are released through the plasma membrane. The initial step of EV formation consists of membrane remodeling via bubble formation and an increase in intracellular calcium levels, resulting in a rearrangement of the phospholipid layer and the exposure of phosphatidylserine. Concomitant with the loss of membrane asymmetry, calcium-sensitive enzymes are activated and promote the cleavage of cytoskeletal filaments, leading to the formation of membrane blebs and the release of EVs ^(9,10)^. EVs arise from various cell types and can be released under the influence of cytokines, thrombin, endotoxins or physical stimuli, as well as shear stress or hypoxia ^(11)^. Moreover, EVs carry markers on their surfaces that enable identification of the cell of origin.

This study aimed to evaluate PS^+^-EVs and EVs related to cardiovascular diseases - EVs from cardiomyocytes, endothelium, platelets and EVs that express tissue factor (TF) - in individuals carrying FH-related genetic variants and to compare them with those in a control group.

## SUBJECTS AND METHODS

### Study population

In this study, 32 individuals were included - 16 FH patients and 16 controls - assisted by the Itinerant Hipercol Brazil program of Instituto do Coração (InCor), Brazil ^(12)^. This program aims to investigate the presence of genetic variants in populations with a high prevalence of dyslipidemia, according to information collected by local medical assistance, with an active search for index cases (ICs) based on medical records and cholesterol tests conducted in laboratories for the clinical analysis of local health units.

**Figure 1:**
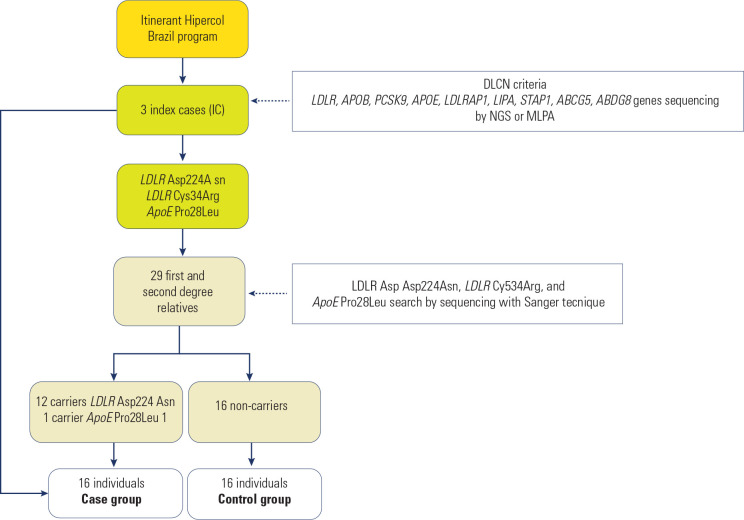
Selection of individuals with Familial Hypercholesterolemia (FH).

Individuals in the case group were diagnosed according to the clinical criteria of the Dutch Lipid Clinic Network (DLCN) ^(13)^. Individuals with scores of > 8 points were diagnosed as definitive FH, those with 6-8 points were diagnosed as probable FH, and those with 3-5 points were diagnosed as possible FH. Those with scores < 3 were considered to have no FH (FH-absent). All ICs presented LDL-c levels ≥ 210 mg/dL (adults) or ≥ 190 mg/dL (patient age: < 18 years). Firstand second-degree relatives without genetic variants in the evaluated genes, who did not meet the DLCN criteria and whose lifestyle and diet were the same as their affected relatives, were included as controls (**Figure 1**). Individuals with hepatic or hematological diseases, descompensated diabetes mellitus (HbA1c > 7.0%), obesity (BMI > 30 kg/m^2^ for adults and BMI Z score > +2 for children), inflammatory and thyroid diseases, and triglyceride levels > 400 mg/dL and who were HIV carriers were excluded from both groups.

From the Itinerant Hipercol Brazil program, three index cases (ICs) were selected based on Dutch criteria and were sequenced in nine genes related to FH by NGS or MLPA. After the genetic variant detected in ICs (Asp224Asn and Cys34Arg in LDLR gene, Pro28Leu in APOE gene), 1^st^ and 2^nd^ degree relatives were included, in a total 29 individuals were sequenced by the Sanger technique to search the variant detected in ICs. 13 individuals carrying the genetic variants (12 in LDLR gene Asp224Asn and one in APOE gene Pro28Leu) and three IC formed the case group; 16 individuals not carrying the genetic variants formed the control group.

Genetic characterization was performed as described in our previous study ^(14)^, with the *LDLR* (Gene ID: 3949), *APOB* (Gene ID: 338), *PCSK9* (Gene ID: 255738), *LDLRAP1* (Gene ID: 26119), *LIPA* (Gene ID: 3988), *STAP1* (Gene ID: 26228), *APOE* (Gene ID: 348), *ABCG5* (Gene ID: 64240) and *ABCG8* (Gene ID: 64241) genes sequenced by performing next-generation sequencing (NGS) of samples from ICs. The samples of individuals for whom no variants were observed through NGS analysis were also subjected to the multiplex ligation probe amplification (MLPA) technique to track copy number variants (CNVs) in the *LDLR* gene. DNA sequencing of family members was performed using the Sanger sequencing method after the patient’s genetic variant was characterized. Thus, those carrying genetic variants in at least one of the investigated genes were considered to have FH and included in the case group.

### Ethical aspects

The present study was approved by the Research Ethics Committee of the UFMG (COEP-UFMG) (CAAE-76387417.6.0000.5149) and by the Ethics Committee of the University of São Paulo (CAAPesqprotocolo100594212.0.1001.0068) and was conducted in accordance with the ethical guidelines of the 1975 Declaration of Helsinki. All participants or their legally acceptable representatives signed written informed consent forms.

### Sample collection and processing

Blood samples (5.0 mL) were collected through venipuncture in sodium citrate (3.2%) tubes in a vacuum system with a maximum tourniquet time of 1 min. Prior to blood collection, patients fasted for 8-12 h. Platelet-poor plasma (PPP) was prepared as follows: 1) the sample was centrifuged at 3,500 rpm for 15 min; 2) the upper 2/3 of the supernatant was transferred to a new tube, and centrifugation was again carried out at 3,500 rpm for 15 min; and 3) only the upper two-thirds of the supernatant were distributed in 500 µL aliquots in polypropylene tubes and stored at -80 °C until the EV assays were conducted.

### Quantification and characterization of extracellular vesicles

EV analysis was performed through flow cytometry as previously described ^(15)^. The antibodies and fluorochromes used in this panel were anti-caveolin-3-Alexa Fluor 647 (cardiomyocytes), anti-CD41a-PE-Cy7 (platelets), and anti-CD142-PE (tissue factor). Annexin V-eFluor450 was used to label EVs that expressed phosphatidylserine (PS) on their surfaces (PS^+^-EVs). The results of the phenotyping panel are summarized in **Table S1 (Supplementary data)**.

Prior to the EV quantification assay, an EV-rich suspension was obtained. The PPP was thawed at 37 °C and centrifuged at 1,300 rpm for 5 min to obtain platelet-free plasma (PFP). Afterward, the supernatant was aspirated and diluted (1:3) in citrate phosphate-buffered saline (PBS) containing heparin (1 µg/mL). This solution was then centrifuged at 15,000 rpm for 90 min at 15 °C (at low deceleration). The resultant EV pellet was then resuspended in 100 µL of 10× annexin V binding buffer^®^ (Thermo Fisher Scientific, San Diego, USA) to produce an EV-rich suspension.

A “fluorescence minus one” control, which is a sample without the labeling of a particular antibody-fluorochrome, was used before the analysis. The labeling and analysis process was designed for multiparametric analysis in a single tube. Aliquots of 100 µL of EV-rich suspension were transferred to polypropylene bottles. The antibody-fluorochromes were subsequently added in the following order: 2.5 µL of Annexin V-eFluor450, 2.0 µL of anti-caveolin-3-AlexaFluor647, 1.0 µL of CD41a-PE-Cy7, and 2.0 µL of CD142-PE. A 30-min incubation (at room temperature) step was performed after each addition of the antibody-fluorochrome, followed by a wash step with PBS-saline, and centrifugation (1,500 rpm for 20 min at 4 °C) was carried out to separate and discard the supernatant. The resuspension was performed with 10× annexin V binding buffer. These critical steps were performed in the dark as much as possible. Three aliquots were used as internal controls: an aliquot containing only the buffer and lacking antibody-fluorochromes and EVs, a second aliquot containing the buffer and Annexin V-eFluor450 and lacking EVs, and a third aliquot containing the buffer and EVs and lacking Annexin V-eFluor450.

**Figure 2:**
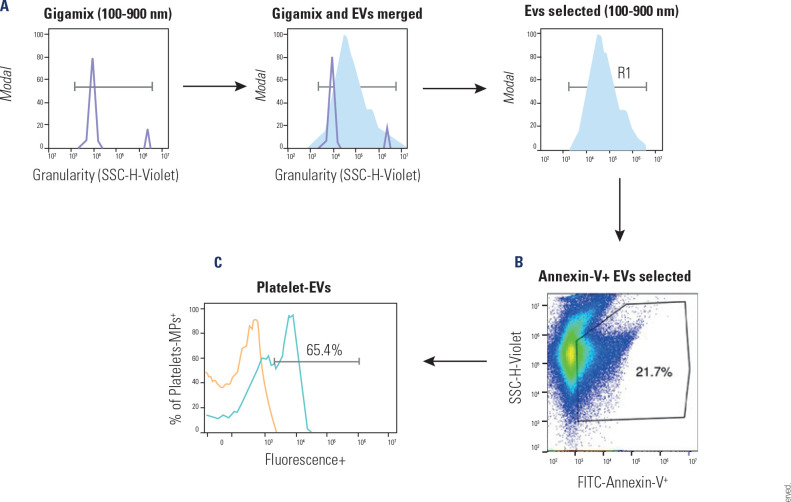
Representative strategies for the Immunophenotyping of Extracellular vesicles (EVs) by flow cytometry.

EVs were detected in a CytoflexS^®^ (Beckman Coulter Inc., Brea, USA) and gated on the basis of their forward (FSC) and side (SSC) scatter distributions of calibration microbeads (Flow Cytometry Submicron Particle Size Reference Kit^®^, Thermo Fisher Scientific), which provide six sizes of green-fluorescent beads: 0.1 µm, 0.2 µm, 0.5 µm, 1.0 µm and 2.0 µm in diameter. The sample flow rate was 30 µL/min, and the cytometer was programmed to operate at a high flow rate for 60 s for each sample (50,000 events). The CytoflexS^®^ has a volumetric sample injection system that allows absolute particle counting. Analysis was performed using FlowJo^®^ software (Tree Star). The gating strategy applied is shown in **Figure 2**.

(**A**) One-dimensional granularity histograms (violet SSC-A) were constructed for the selection of gigamix beads ranging from 100 to 900 nm. Merging one-dimensional Gigamix and MP histograms allow the selection of EVs from 100 to 900 nm (R1). (**B**) Two-dimensional plot of FITC-Annexin V vs. granularity (SSC-H-Violet) with EVs (100 and 900 nm) selected the positivity region for FITC-Annexin V. (**C**) Histograms are used for the quantification of the percentage of phenotype-specific EVs in the samples.

### LDL-c and lipoprotein(a) (Lp(a)) quantification

The LDL-c values, obtained from medical records, were evaluated under individual baseline conditions, that is, without treatment. However, in FH patients receiving lipid-lowering treatment (13 patients from the case group), baseline LDL-c was estimated using conversion factors reported in the literature according to Foody and cols. ^(16)^ and Ballantyne and cols. ^(17)^.

Serum Lp(a) was quantified using the Atellica CH^®^ diagnostic kit, which is based on the turbidimetry method, and an Atellica Siemens Healthineers^®^ analyzer.

### Statistical analysis

To calculate the sample size, we considered a type I error (α) of 0.05 and a type II error (β) of 0.20 (80% power). On the basis of a pilot analysis, we adopted a standardized effect magnitude of 0.8, considering the number of PS^+^-EVs as the main variable. On this basis, 15 patients were required in each group. Statistical analysis was performed using the R Platform version 4.2.2 program. Qualitative variables are described as absolute and relative frequencies of their categories, and quantitative variables are described as measures of central tendency (medians and interquartile ranges (IQRs), or means ± standard deviations). The normality test applied was the Shapiro-Wilk test. The association between qualitative variables was tested using the chi-square test, with p values calculated via Monte Carlo simulation when necessary. Comparisons of the central tendency of quantitative variables between groups were performed using Student’s *t* test or the Mann-Whitney test for the two groups. Correlation analysis was performed using the Spearman test. The significance level adopted was 5%, with a statistical power of 95%.

## RESULTS

The case group consisted of 14 individuals carrying genetic variants in the *LDLR* gene (13 with Asp224Asn, a pathogenic variant; and one with Cys34Arg, a probable pathogenic variant) and two individuals with genetic variants in the *APOE* gene (both carrying Pro28Leu, a variant with uncertain significance) ^(14)^. The other 16 relatives were not carriers of these genetic variants and were classified into the control group.

Carriers of genetic variants presented with a corneal arch (12%), xanthoma or xanthelasma (23%), and these characteristics were not observed in noncarriers. Age, Lp(a) level, sex, frequency of diabetes mellitus, systemic arterial hypertension, history of cardiovascular disease and smoking status did not significantly differ between the groups (p > 0.05 for all). However, body mass index (BMI) was higher in the group carrying genetic variants, and LDL-c levels, as expected, were also higher in this group, as the LDL-c level is a diagnostic criterion for FH (p = 0.025 and p < 0.001, respectively). The clinical and demographic characteristics of the case and control groups are summarized in **Table 1**.

**Table 1:** Clinical and demographic characterization of carrier and non-carrier groups of genetic variants related to familial hypercholesterolemia

Variables	Non-carriers (n = 16)	Carriers (n = 16)	P value
LDL-c (mmol/L)	3.6 ± 0.9	7.6 ± 2.1	<0.001^*^
Lp(a) (mmol/L)	0.3 (0.2-0.57)	0.5 (0.2-1.24)	0.216
Age (years)	31.9 ± 21.7	43.6 ± 12.2	0.064
Gender (%) Male Female	20 80	24 76	0.810
BMI (kg/m^2)^	21.7 ± 5.5	26.3± 5.2	0.025^*^
Corneal arch (%)	0	12	-
Xanthomas/Xanthelasma (%)	0	23	-
CVD history (%)	6.7	5.9	0.927
Diabetes mellitus (%)	0	0	1.000
Hypertension (%)	13.3	23.5	0.659
Smoking (%) Non-smokers Ex-smokers# Smokers	100 0 0	88.2 5.9 5.9	0.390

Continuous variables expressed as median (interquartile range - 25^th^-75^th)^ or mean ± standard deviation, categorical variables expressed as frequency. Data not normally distributed were compared by Mann-Whitney U test. Data normally distributed were compared by Student T-test.

BMI: Body Mass Index; CVD: cardiovascular disease.

* Significant p < 0.05. #Up to 1 year before collection.

EVs: extracellular vesicles; PS+-EVs: EVs marked with AnnexinV-eFluor450; CardioEVs: EVs derived from cardiomyocytes; EEVs: EVs derived from endothelial cells; TFEVs: EVs that express tissue factor in surface; PEVs: EVs derived from platelets. Data not normally distributed were expressed as median (25^th^-75^th)^ and compared by Mann-Whitney U test.

* Significant p < 0.05.

**Figure 3:**
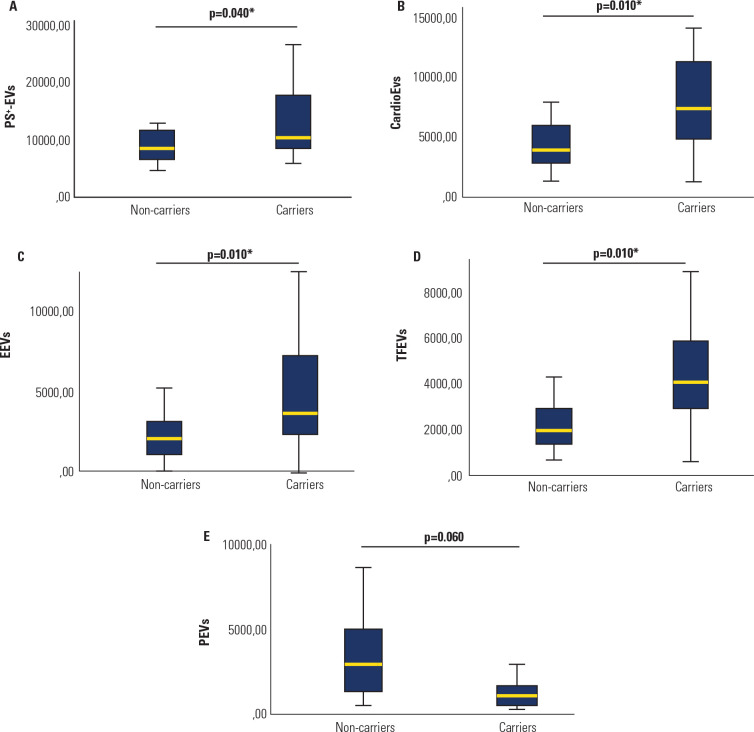
Comparison of extracellular vesicles count (EVs/µL) between carriers and non-carriers of genetic variants related to Familial Hypercholesterolemia. **A**) PS^+^-EVs, **B**) CardioEVs, **C**) EEVs, **D**) TFEVs, **E**) PEVs.

Patients with FH who carried genetic variants had higher counts of PS^+^-EVs (p = 0.040; **Figure 3A**), CardioEVs (p = 0.010; **Figure 3B**), EEVs (p = 0.010; **Figure 3C**) and TFEVs (p = 0.010; **Figure 3D**) than family members without FH and noncarriers did, regardless of age or sex. No significant difference in the number of PEVs was detected between the groups (p = 0.060; **Figure 3E**).

Age was not correlated with EV counts. In contrast, BMI was weakly correlated with CardioEV counts (p = 0.352, p = 0.048). A significant and positive correlation was observed between LDL-c levels and counts of PS^+^-EVs (p = 0.500; p = 0.004), CardioEVs (p = 0.509; p = 0.030), EEVs (p = 0.618; p < 0.001) and TFEVs (p = 0.623; p < 0.001) in both groups. No correlation was observed between the PEV count and LDL-c level (p = -0.326; p = 0.068). Interestingly, the PS^+^-EV count was also significantly correlated with Lp(a) levels (p = 0.360; p = 0.043).

**Figure 4:**
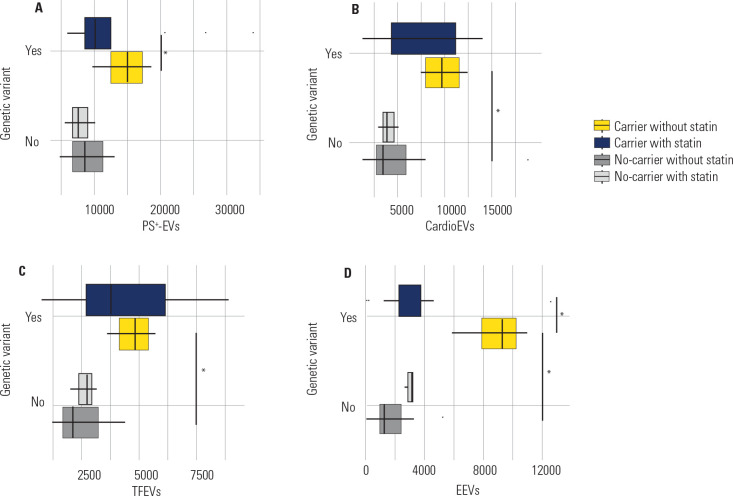
Comparison of extracellular vesicles count (EVs/µL) between carriers and non-carriers of genetic variants according to statin use. **A)** PS^+^-EVs: EVs marked with AnnexinV-eFluor450; **B)** CardioEVs: EVs derived from cardiomyocytes; **C)** TFEVs: EVs that express tissue factor in surface; **D)** EEVs: EVs derived from endothelial cells. *Significant p < 0.05.

Higher counts of total PS+-EVs were observed in the group of individuals with FH who did not use statins than in individuals without FH and who did not use these drugs (p = 0.020) (**Figure 4A**). Similarly, higher CardioEV and TFEV counts were observed in the group of individuals with FH than in the group of individuals without FH, both of whom did not use statins (p = 0.020 for both) (**Figures 4B and 4C**). Individuals with FH had higher EEV counts than individuals without FH did; neither group used statins (p < 0.001) (**Figure 4D**). Additionally, in the group of variant carriers with FH, individuals who were not using statins had higher EEV counts than those who were using this drug did (p = 0.010) (**Figure 4D**). PEV counts did not significantly differ between carriers of genetic variants with FH and noncarriers because of the use of statins (p > 0.050 for all comparisons; data not shown).

## DISCUSSION

In this study, 32 individuals were evaluated, 16 of whom were carriers of genetic variants related to FH, and 16 of whom were 1^st^- and 2^nd^-degree family members who were not carriers of the variants found in family clusters. Higher numbers of PS^+^-EVs, CardioEVs, EEVs and TFEVs were observed in the group with FH than in the group without FH. In addition, higher counts were observed among individuals with FH who did not use statins, which suggests that these drugs may play a regulatory role in the release of EVs, either by directly reducing cell activation or injury or by reducing LDL-c levels. In fact, EV counts were positively correlated with LDL-c levels. It is also important to emphasize the correlation of PS^+^-EVs with Lp(a), a marker that is related to FH ^(18)^.

Hypercholesterolemia is related to increased oxidative stress. This oxidative stress predisposes individuals to increased accumulation of oxidized LDL cholesterol (oxLDL-C), which interacts with the CD36 receptor. Nielsen and cols. ^(19)^ reported that elevated oxLDL-C levels induce proinflammatory monocytes and increase the release of monocyte-derived EVs in subjects with heterozygous FH, particularly in the presence of Achilles tendon xanthomas (ATX). The same group reported that total microvesicles (MVs) and monocyte-, endothelial-, erythrocyte-, and tissue factor-positive cell-derived MVs were significantly greater in FH patients than in controls. In addition, counts of CD36+ MVs derived from endothelial cells and monocytes were significantly higher in FH patients, and oxLDL-C levels predicted all the investigated cell-specific CD36+ MVs in FH patients with ATX ^(20)^.

Chen and cols. ^(21)^ analyzed the role of PS^+^-EVs in the atherosclerotic process and suggested that they may induce endothelial dysfunction, vascular inflammation, coagulation, thrombosis, and calcification through their protein components and noncoding RNAs, which may promote atherosclerosis. EVs may represent important pathways of intercellular communication and act as messengers, accelerating the atherosclerosis process, and may become diagnostic biomarkers in the treatment of atherosclerotic disease. In fact, a greater number of EVs with reduced expression of microRNAs, related to protective factors, was demonstrated in human coronary artery smooth muscle cells from patients with FH than in those from control individuals ^(22)^.

When EVs were characterized according to cell origin, we observed that the number of CardioEVs was significantly greater in the FH group than in the non-FH group. To the best of our knowledge, this is the first study to evaluate the relationship between CardioEVs and FH. Although cardiomyocytes are not considered typical secretory cells, their EVs can be released because of cellular activation, hypoxia, apoptosis, injury and inflammation, conditions that cooccur with coronary artery disease early in individuals with FH ^(23,24)^. A recent study from our group revealed that the number of cardiomyocyte-derived EVs was relatively greater in patients with breast cancer who developed cardiotoxicity secondary to doxorubicin chemotherapy. A positive correlation with serum NT-proBNP levels was also found, which suggests that CardioEVs are involved in the pathophysiology of cardiac cell injury ^(25)^.

EEV counts were higher in the group of individuals with FH than in individuals without FH. In addition, in individuals with FH, the number of nonusers of statins was greater than that in individuals with FH and those using statins, corroborating the hypothesis that statins reduce endothelial damage and, consequently, the release of EEVs. EVs originating from endothelial cells are suggestive of vascular pathologies, as they are cellular responses to an activated, compromised and damaged endothelium. Therefore, EEVs are a predictive marker of vascular health ^(26)^. Studies have shown a significant increase in the number of circulating EEVs in association with coronary artery disease, suggesting that this elevation is not only related to coronary endothelial dysfunction but is also associated with an increased risk of major cardiovascular events ^(27,28)^. Interestingly, EEVs can also promote cell survival, exert anti-inflammatory effects, counteract coagulation processes, or induce endothelial regeneration ^(29)^.

The number of EVs carrying cellular markers of different circulating cellular origins (platelets, endothelial cells, and leukocytes) was significantly lower in hypercholesterolemic patients on statin-based lipid-lowering therapy than in untreated hypercholesterolemic patients, in addition to a reduction in the number of platelets, activated inflammatory cells and tissue factor ^(30)^. Furthermore, the effect of lipid-lowering therapy on EV clearance is cumulative over time, and patients receiving statin treatment had significantly lower numbers of EVs carrying activated cell markers, indicating that statins protect against cell activation-related vascular disorders ^(30)^. Another study comparing two groups, one in which atorvastatin 10 mg was used and the other in which atorvastatin 40 mg was used, revealed a reduction in the number of circulating EEVs and an increase in the number of circulating endothelial cell progenitor cells in patients with ischemic cardiomyopathy compared with those in the group in which atorvastatin 10 mg was used. The effect was independent of the decrease in lipids, LDLox and ultrasensitive C-reactive protein (us-CRP) ^(31)^, indicating that the benefit of statin treatment in FH patients goes beyond its effect on lowering LDL-c levels.

In the analysis of EVs that express tissue factor (TFEVs), higher counts were observed in individuals with FH who carry genetic variants. Tissue factor is highly expressed in atherosclerotic plaques, and it is upregulated by LDLox ^(32)^. By analyzing TFEVs in relation to lipid-lowering treatment with statins in an animal model, a study was conducted to assess whether simvastatin can inhibit tissue factor production in monocytes from hypercholesterolemic animals, considering that hypercholesterolemia can produce a procoagulant state. Simvastatin treatment reduced LDLox levels and, consequently, decreased monocyte tissue factor expression and TFEV activity, reducing the prothrombotic state ^(33)^.

Cardiomyocytes and cardiac fibroblasts constitutively express high levels of TF. Beyond its role in maintaining systemic hemostatic balance, TF is essential for cardiac hemostasis and acts as a protective barrier against intracardiac bleeding and hemorrhage. Experimental models with reduced TF expression have increased mortality, likely because extensive fibrosis predisposes patients to malignant arrhythmias and sudden death. Furthermore, TF expression and activity are markedly elevated in the myocardium following ischemia-reperfusion injury ^(34)^. These findings corroborate our results, in which TFEV and CardioEV counts were both increased in the FH group, suggesting a physiological relationship.

In the evaluation of PEVs, no significant difference was observed between the groups regarding the presence of FH/genetic variants. Platelets play a central role in primary hemostasis because of aggregation and secondary hemostasis because of their procoagulant properties ^(35)^. The FH patients in our study were young (mean age of 43.6 years) and possibly had incipient thrombus in atheromatous plaques, a condition observed in patients with more advanced processes.

This study has several limitations, mainly the limited sample size. In addition, genetic variants in other uninvestigated genes related to LDL metabolism may be present in family members classified as noncarriers. Moreover, characterization using other atherosclerotic biomarkers, such as pulse wave velocity, carotid and femoral plaques, and coronary artery calcification, is lacking. Considering that BMI was correlated with CardioEVs, other studies should investigate the mechanism related to these variables. Therefore, only 1^st^- and 2^nd^-degree relatives were included as controls, which may lead to the underestimation of differences and limit generalizability.

Statin use by FH patients also affects the expression of molecules on EVs, in addition to their production. Mobarrez and cols. ^(36)^ reported that atorvastatin reduces thrombin generation and the expression of TF, glycoprotein IIIa and P-selectin on PEVs in patients with peripheral vascular disease. In agreement with these findings, Tehrani and cols. ^(37)^ reported reduced expression of glycoprotein IIIa, P-selectin and TF on PEVs in patients with type 1 diabetes mellitus and dyslipidemia. Finally, Almquist and cols. ^(38)^ reported that simvastatin reduced the expression of P-selectin, TF and CD40L on PEVs and of TF on monocyte-derived EVs in patients with diabetes mellitus and chronic kidney disease. Consequently, future studies including FH patients before statin treatment should be conducted to evaluate the effect of genetic variants related to FH on EV count.

In conclusion, EVs have increasingly been recognized as promising candidates for both diagnostic and therapeutic applications. Beyond their potential utility as biomarkers of pathological processes and as vehicles for targeted interventions, EVs may further contribute to elucidating the impact of diverse confounding variables on disease mechanisms and clinical outcomes in FH patients. Our findings suggest that EVs are related to FH, since the counts of PS^+^-EVs, cardio-EVs, EEVs, and TFEVs, which are involved in the atherosclerosis process, were greater in individuals with FH and may be related to cardiovascular outcomes in these patients. However, considering the limitations of our study, we consider that these preliminary results should be validated in a large population with different lifestyles and genetic characteristics.

## Data Availability

datasets related to this article will be available upon request to the corresponding author.

## SUPPLEMENTARY MATERIAL

**Table S1:** Extracellular vesicle phenotyping panel

Filter	530/30	575/26	780/60	670/14	450/50
Antibody	anti-CD51/61	anti-CD142	anti-CD41a	anti-caveolin-3	Annexin V
Fluorochrome	FITC	PE	PE-Cy7	Alexa Fluor 647	eFluor 450
Specificity	endothelium	tissue factor	platelets	cardiomyocyte	phosphatidylserine
Concentration	1.0 µg/100 µL	0.125 µg/100 µL	0.125 µg/100 µL	1.8 µg/100 µL	5.0 µL/test
Clone	monoclonal (clone 23C6)	monoclonal (clone HTF-1)	monoclonal (clone HIP8)	polyclonal	monoclonal
Catalog number#	11-0519-42 (ThermoFisher)	12-1429-42 (ThermoFisher)	25-0419-42 (ThermoFisher)	sc-5310 (Santa Cruz)	88-8006-72 (eBioscience)
